# Role of autoimmunity in Neuronal damage in children with Autism spectrum disorder

**DOI:** 10.12669/pjms.39.6.6804

**Published:** 2023

**Authors:** Dost Muhammad Halepoto, Laila Yousif AL-Ayadhi, Abdulrahman Mohammed Alhowikan, Nadra Elyass Elamin

**Affiliations:** 1 Dr. Dost Muhammad Halepoto, PhD. Autism Research and Treatment center, Department of Physiology, Faculty of Medicine, King Saud University, Riyadh, Saudi Arabia; 2 Dr. Laila Yousif AL-Ayadhi, PhD. Autism Research and Treatment center, Department of Physiology, Faculty of Medicine, King Saud University, Riyadh, Saudi Arabia; 3 Dr. Abdulrahman Mohammed Alhowikan, PhD. Department of Physiology, Faculty of Medicine, King Saud University, Riyadh, Saudi Arabia; 4 Dr. Nadra Elyass Elamin, PhD. Autism Research and Treatment Center, Department of Physiology, Faculty of Medicine, King Saud University, Riyadh, Saudi Arabia

**Keywords:** Autism spectrum disorder, Autoimmunity, Neuroinflamation, Central nervous system

## Abstract

“Autism spectrum disorder (ASD) is complex neurodevelopmental disorder characterized by impairments in three core behavioral: social deficits, impaired communication, and repetitive behaviors.” There is developing indication and emerging data that irregular autoimmune responses to the central nervous system may play a pathogenic role in patients with autism spectrum disorder.”

The aim of this review was to discuss the updated research carried out at Autism research and treatment center, King Saud University, Riyadh, Kingdom of Saudi Arabia particularly on the role of autoimmunity in Autism spectrum disorder. This review also present state of information available about the role of autoimmunity biomarkers involved in the neuronal damage of central nervous system in autistic children. The systematic literature search was carried out using Google Scholar, Science direct and PubMed databases on the role of autoimmunity in autism and reviewed all relevant articles published in peer reviewed journals by Autism research and treatment center, King Saud University, Riyadh, Kingdom of Saudi Arabia till April, 2022. We searched relevant articles using key words Autism spectrum disorder, Autoimmunity, Neuroinflamation and Central nervous system. This review revealed that plasma levels of autoimmunity related factors/ markers were altered in patients with autism. Significant change in blood markers in subjects with ASD may resulted in several years of decreased neutrotrophic support along with increasing impairment in relationship with down-regulated inflammation that may play a role in the ASD.

Overall, the role of autoimmunity in ASD subjects with excess of anti-brain antibodies suggest that in some patients, autoantibodies that target the CNS may be pathological factor in neuronal growth in autistic children. Large cohort studies with well-defined and specially pheno typed autistic groups and matched healthy controls are required to examine the role of autoantibodies in the pathology of subjects with ASD.

## INTRODUCTION

Autism spectrum disorder (ASD) is a neurodevelopmental disorder characterized by deficits in social communication and the presence of restricted interests and repetitive behaviors.^1^ The disorder manifests during the age of early three years and continues into later life. There is developing indication that irregular immune responses play a major role in the pathophysiology of ASD.^2^

Overall, the links among the neurological and immunological systems are very important in various neurological conditions. Sleep and mood behaviors can be influenced by several cytokines and other products of immune activation, because of prevailing consequences on neurons.^3^ Abnormal immune action throughout the early critical periods of brain and neuronal development could possibly play a role in neuronal dysfunction. Numerous research investigations^2^ have demonstrated defects of the immune reaction in ASD, along with irregular production of cytokines, immune cells and antibodies.

### Neurological defects in ASD:

In the early age neural brain growth (migration, neuronal differentiation, synapse formation and axonal extension) takes place. Several neurological irregularities have been established in ASD which proposed that usual neurodevelopment was interrupted in an acute period of brain growth.^4^ Twenty five to thirty percent (25-30 %) of autistic children (aged 18-22 months) undergo a period of autistic deterioration, in which they go through loss of formerly developed language and social skills.^5^ Many reports have suggested that several main brain structures may be disturbed in ASD.^6^ Moreover, brain areas included in ASD have a tendency to progress gradually and are more likely to disruptions. Cerebellar deformities have been identified as the utmost common outcome in ASD, directing in specific granular and Purkinje cells.^7^

ASD results from over-pruning of brain connectivity early in development, particularly effecting long-range connections. Resting state studies demonstrate significantly reduced functional connectivity within and between resting state networks incorporating ‘social’ brain regions. This reduced connectivity may result in difficulties in social interaction and communication, which is reflected in hypoactivation of amygdala in ASD individuals.^6^ Furthermore, the amygdala volume of young ASD children is significantly enlarged and comprised a significantly lower number of neurons relative to that of young healthy children.^6^

Moreover to immune genes mutations, autoimmune disorders are highly related with the families of autistic children.^8^ Research studies have demonstrated that there is maximum fifty percent rise in the chances of ASD diagnosis among children or parents who have an autoimmune disorder.^8^ Certainly, autoimmune disorders are over-represented in the ASD people.^9^ Though, such disorders also raise the chance of several neurodevelopmental disorders including intellectual disability, suggest that immune disorders usually increase the susceptibility of the developing brain to progressive deficiencies.^10^

### Immunological innovations in ASD:

Autoimmunity is a disease resulted when the body produce an unsuitable immune reaction against its own tissues. Occasionally the immune system is ended to categorize one or more of the body’s usual components as “self” and creates autoantibodies that attack its own cells, tissues, and/or organs. This results damage and inflammation which leads to autoimmunity.

In 2011 Al-Ayadhi explained 3 that the exact mechanism of autoimmunity in autoimmune diseases is not similar, but they all have auto reactive antibodies and T cells. Moreover, the existence of antibodies directed against components of the central nervous system (CNS) in the sera of autistic children is indicative of an autoimmune process that may be involved in the pathology of some cases of ASD.

Mostly with all autoimmune diseases, environmental, immunological and genetic, factors, play major roles in the progress of the disease.^11^ Food sources containing gluten, casomorphins, casein, and gluteomorphins, the opioid peptides, are linked with the pathogenesis of ASD.

Baspinar and Yardimci investigated^12^ the effect of Gluten-Free Casein-Free diet to solve the gastrointestinal and behavioral problems in ASD. Incompletely digested peptides, toxins, and proinflammatory cytokines enter in the bloodstream and reach the central nervous system which adversely affect the brain function. In addition to nutritional problems various uncontrolled immune responses arise in individuals with ASD, including increased natural killer cell activity, presence of autoantibodies directed to brain proteins, and modified cytokine profiles, which cause several diseases associated with ASD^12^.

Immunoglobulin’s (IgG, IgM and IgA) were identified, against nine specific neuron-specific antigens in the blood sera of autistic children.^13^ These resulted antibodies were attached with different CNS molecules that have sequence homologies to a milk protein. The occurrence of brain-specific auto-antibodies in some autistic children^14^ indicated that autoimmunity may play a role in the etiology of ASD. Moreover, there is also an upsurge in the rate of autoimmune disorders among ASD families.^14^ Despite the fact that the cause of autoimmunity in ASD is not known, involvement of the major histocompatibility complex genes and their products cannot be ruled out.^15^ Though, at present, the indication for contribution of the immune system in ASD is still not clear. However immune system irregularities have been described in autistic children, nevertheless, their function in central nervous system is less known.

### Neurogenic inflammation in autoimmune diseases:

Neurogenic inflammation is caused due to the release of neuropeptides containing tachykinins (neurokinin B, substance P and neurokinin A) from the exhibiting part of the noncholinergic, nonadrenergic excitative nervous system after exposure to allergens.^16^ Neuropeptides, such as neurotrophins and tackykinins have been believed as fundamental intermediaries of neuro-immune associations in some autoimmune disorders.^17^ It is also reported that neuropeptides play a potential role in some autoimmune neuroinflammatory and systemic autoimmune diseases.^18^

Suggestion for relation between neural dysfunction points and chronic inflammation in autoimmune diseases to an involvement in the immune and the nervous system is established. Some investigations have suggested that antibodies may act as markers for specific disease expressions, including CNS disease.^19^ The maintenance and development of the CNS is effected by several different factors, one of the most important being neurotrophic factors (NTFs).^20^ Moreover, individual NTFs and their receptors are comparatively different and are subject to significant variations in the neural growth.^21^

Autoimmunity may be a major issue in the etiology of ASD. A dynamic factor of the autoimmune route should contain antibodies against brain, the concerned tissue in ASD. In this relation, a few studies in ASD have confirmed indication of antibodies to brain tissue antigens, e.g., serotonin receptor, neurofilament proteins and Myelin basic protein (MBP).^22^ Moreover,the proinflammatory cytokines such as interleukin (IL)-1, IL-6, IL-12, interferon-(IFN) and tumor necrosis factor (TNF)” have been exposed and directly affect neural tissue growth and function. 23

### Possible neuroimmune mechanism: development of autism:

There is developing concentration on deciding by what means immune dysregulation might change function and brain connectivity to play role in ASD. Meanwhile, in the brain immune molecules associate with glia or neurons have been known to control each stage of function and growth of brain.^24^ The body reuses immune molecules, immune cells, and their receptors for extensively different assignments in both area and advanced phase certain means. Modifications in the expression of these immune molecules in the brain, as an outcome of environmental risk factors or by genetic mutations can lead to temporary and/or permanent modifications in brain function and growth.

It was revealed that irregularities in immunity, may play a part in brain synaptic functions and development hereafter in the pathophysiology of ASD.^25^ Cytokines such as interleukin-6 (IL-6), interleukin-4 (IL-4), Interferon gamma (IFN-γ), Transforming growth factor β (TGF- β1) and Tumor necrosis factor-α (TNF-α) influence directly on neurons function. They are involved in neurodevelopment in prenatal and postnatal period.^26^ There is also a group of indication involving cytokines to greater order neurological roles, including memory and cognition.^27^ Hence, interruption within cytokine homeostasis may be liable for a range of neurological outcomes associated to ASD^25^.

In the nervous system interleukin-1 beta (IL-1β) is present during plasticity, synapse formation, differentiation, migration, neurogenesis and responses to injury.^28^ Earlier study also verified that permanent expression of IL-1β in hippocampus impairs spatial memory.^29^ The IL-1β has been also play role in neural progenitor cell proliferation in specific CNS areas which provide to region-particular progress in the brain of autistic subjects.^25^ The deficiency of the protein expression outcomes in altered CNS development may lead to motor incoordination, seizures, and severe behavioral abnormalities.^30^

Keeping in view of these facts, the objective of this study was to review the literature reported by ARTC, KSU, Riyadh, KSA with respect to autoimmune phenomena and possible association with damage of neurons in CNS in children with ASD.

## METHODS

### Search Strategy:

All relevant articles in English language on the role of autoimmunity markers in ASD published in peer reviewed journals by Autism research and treatment center (ARTC), King Saud University (KSU), Riyadh, Kingdom of Saudi Arabia (KSA) were reviewed, till 2022. A systematic electronic search was conducted for records indexed within Science Direct, Google Scholar, and PubMed, or Web of Science. Within these databases, we identified thirty-one (31) relevant articles using the following key words Autism spectrum disorder, Autoimmunity, Neuroinflamation and Central nervous system. The published articles were first selected by titles, then by abstracts, followed by assessment of full-text.

### Selection criteria:

Selection criteria of primary studies were as follows: (1) carried out with blood samples of subjects diagnosed with ASD according to Diagnostic and Statistical Manual of Mental Disorders (DSM V); 1 (2) measured autoimmunity related markers; (3) compared with matched controls. (4) Articles were excluded that did not meet inclusion criteria.

### Data Extraction:

Two authors independently extracted data from all retrieved articles using a standardized data collection form. Information extracted included full reference with publication year, autoimmunity markers, number of subjects with age, values obtained with results/outcome.

### Study Characteristics:

The main characteristics and findings of some autoimmunity related studies in Saudi Children with ASD published by ARTC, KSU, Riyadh, KSA are summarized in Table-I. The included studies were published at varying times, ranging from 2011 to 2022, the included studies adopted different methodological approaches [Enzyme-linked immunosorbent assays (ELISA) Indirect immunofluorescence (IF), Quantitative sandwich enzyme immunoassay (QSEIT), Polymerase chain reaction (PCR) and Buhlmann titre unit (BUT)] to evaluate autoimmunity markers in serum/plasma of ASD subjects.

**Table-I:** List of some studies on role of autoimmunity related Proteins/Antibodies in children with ASD, published by ARTC, King Saud University, Riyadh, KSA.

Protein/Antibody	Age in years, A= autistic, C= control	Method/Source	Values	Outcome/Result	Ref
Antineuronal antibodies	6-12, A=80,C=80	IIF/S	A=62.5% C=5%	Autistic has higher positivity than healthy controls	2
Anti-ds-DNA antibodie Anti-nuclear Antibodies (ANA)	4-11 A=100 C=100	ELISA/S	34% and 25%	Increased respectively in autistic children	31
Antiendothelial cell antibodies (AECAs)	3-12 A=55,C=25	ELISA/S	(mean ± SEM) A=306.4 ± 45.6 pg/ml C=209.6 ± 24.6 pg/ml	Increased in A	32
Epithelial cell-derived neutrophil-activating peptide-78 (ENA-78)	4-11 years A=62, C=62	ELISA/S	median (IQR) A=186.26 (105) pg/ml C=112.5 (115) pg/ml	Increased in A	33
Neurokinin A	4 – 12 A=70,C=48	ELISA/S	median (IQR) (pg/ml A= 130 (328) C= 52.5 (31)	Increased in A	16
Anti-Ribosomal P protein	4 – 12 A=70,C=48	ELISA/S	median (IQR) U/ml A= 115 (467) C= 23.5 (248)	Increased in A	16
Progranulin	3 – 12 A=40,C=40	ELISA/S	median(IQR) ng/ml, A=77.50 ±19 C=87.00 ± 81	Decreased in A	34
Serotonin,	5 – 12 A=50,C=30	ELISA/S	median (IQR) ng/ml, A= 243.5 ±119 C=41 ±31	Increased in A	35
Anti-myelin basic protein (MBP)	5 – 12 A=50,C=30		median (IQR) pg/ml, A=465.5 ±348, C= 92 ±49	Increased in A	35
Anti-myelin basic protein (anti-MBP) antibodies	4-12 A=80, C=80	ELISA/S	75%	Increased in A	36
Osteopontin	3 – 11 A=42,C=42	ELISA/S	(mean ± SD) ng/ml, A= 197.10 ± 48.84 C= 122.30 ± 39.16	Increased in A	37
Anti-ganglioside M1	4 – 12 Years, A=54,C=54	ELISA/S	Median (IQR) ng/ml, A= 422 (486) C= 43 (75)	Increased in A	38
S100B protein	4 – 12 Years, A=64,C=46	ELISA/S	mean± SD, pg/ml, A=207.97 ± 52.6 C=171.33 ± 34.65	Increased in A	39
Antiribosomal P protein antibodies			median (IQR) 400 (459) U/ml	Increased in A	39
Anti-myelin-associated glycoprotein (MAG) antibodies	5-12 years A=50, C=30	ELISA/S	mean ± SD A=2274.22 ± 578.32 BTU C=1468.57 ± 302.89 BTU	Increased in A	40
Interleukin-17A	6-11 A=45,C=40	ELISA/S	median (IQR) pg/ml, mean ± SD A=1.7 ±0.9 C=0.77 (0.8)	Increased in A	41
Thymus and activation-regulated chemokine (TARC).	4-12 Years, A=56, C=32	QSEIT/S	median (IQR) A = 880.5 (1206) pg/ml), C= 137.25 (173) pg/ml) pg/ml),	Increased in A	42
Macrophage-derived chemokine (MDC)	4-12 Years, A=56, C=32	QSEIT/S	median (IQR) pg/ml) A= 1289.5 (1079) C= = 190 (295)	Increased in A	42
Antinucleosome-specific antibody	3-12 A=60, C=60	ELISA/S	median (IQR) A=2.9 (2.6) C=1.7 (1)	Increased in A	43
Human leukocyte antigen HLA-DRB1*11	5-12 A=100, C=100	PCR	33/180 6/160	Autistic had higher frequency than controls	44
Human leukocyte antigen HLA-DRB1*03	5-12 A=100, C=100	PCR	40/160 16/180	Autistic had lower frequency than controls	44

A = Autistic, C=Control, IIF = Indirect immunofluorescence technique, S = Serum, P= Plasma, ELISA = Enzyme-Linked Immunosorbent Assay, QSEIT = Quantitative sandwich enzyme immunoassay technique, PCR = Polymerase chain reaction, BTU = Buhlmann titre unit.

## DISCUSSION

This review identified thirty-one (31) studies conducted at ARTC, KSU, Riyadh, KSA. Results revealed that plasma levels of autoimmunity related factors/ markers were altered in patients with ASD. Significant changes in blood markers may resulted in several years of decreased neutrotrophic support along with increasing impairment in relationship with down-regulated inflammation that may play a role in the ASD.

The neurological source for ASD is still not clear, and the association between autoimmunity and neuro-inflammation needs to be discovered.^45^ Research in neurobiological related area in ASD has emphasized trails associated in cognition, behavior, synapse plasticity, neural growth and, structural brain irregularities. Simultaneously, numerous lines of indication direct to changed immune dysfunction in ASD that influences specific or all these neurological routes.^46^

General modifications in immune role have now been defined in both adults and children with ASD, along with continuing inflammation in brain samples, raised pro-inflammatory cytokine levels in the CSF and blood, elevated existence of brain-specific auto-antibodies and reformed immune cell function. Moreover, these dysfunctional immune reactions are linked with amplified impairments in behaviors typical of key characteristics of ASD, in specific, deficits in social interactions and communication. This collective evidence recommends that immune progressions play a significant role in the pathophysiology of ASD.^46^

Previous studies also suggested that autoimmunity play key pathogenic roll to CNS in ASD.^34^, ^37^ This may be designated by the existence of brain-specific auto-antibodies in autistic children.^14^, ^35^, ^38^ Whereas considerably advanced values of autoantibodies were identified in ASD subjects as compared to controls. However, the pathophysiological importance of these antibodies is unclear. All over, the results of autoimmunity in families and the extra anti-brain antibodies propose that in certain ASD subjects, autoantibodies that attack the CNS may be an aggravating or pathological issue in neuronal growth in ASD children. It is also uncertain whether individual children with ASD are positive for more than one antibody. Furthermore, possibly higher autoimmunity may be limited to only a subset of ASD patients.

In fact, detailed cohort studies with systematically described and precisely pheno-typed groups with autism and healthy controls are required to validate the role of autoantibodies in the pathology of autistic children. As for as anti-brain autoantibodies are concerned they were also confirmed for individuals with neurological disorders except autism, and healthy subjects. This is doubt for disease-specificity and pathogenic importance of the antibodies confirmed in the serum of patients with autism.

The cause behind the development of certain autoantibodies in patients with ASD is not well known. It is hypothesized that cross-reacting antigens in the environment might trigger an autoimmune reaction, which release some self-antigens. Such antigens may be produced in the generation of autoimmune reactions via the activation of inflammatory cells in hereditarily vulnerable subjects.^47^ Possible neuroimmune mechanism leading development of autism was described previously.^48^ According to this hypothesis during prenatal period, the body is exposed to infectious agents, which mimic neuron-specific antigens. Following sequence of various events in CNS neurofilaments produce MBP, α-β-crystalline, MAG and MOG, and other antigens.

These antigens enter into the blood circulatory system result in immune responses; leading to create plasma cells that are able to form IgA, IgG, and IgM antibodies against neuron-specific antigens. Antibodies linked with neurons may pass the blood-brain barrier and form immune complexes by combining with brain tissue antigens, thus cause more damage to nervous system tissues. Neuron-specific antibodies, accompanied by toxic biological factors, such as free radicals and arachidonic acid, can also remove neuron myelin and consequently impair electrical conduction e.g. between CNS and muscles. This assumption may describe substantial variations in the values of pathogenic anti-neurological autoantibodies between controls and individuals exposed to toxic chemicals and metals.^49^

It has been also believed that autoimmune disease is generally associated to some genes that regulate immune reactions and ASD is probably an autoimmune response against the brain.^50^ In fact ASD patients react positively to therapy with immune modulating drugs supports the hypothesis that ASD is an autoimmune disorder.^51^

The fundamental basis of immune abnormalities in patients with ASD may range from maternal immune activation to genetic or any different unidentified reasons. Research studies at ARTC so far, suggested an extensive ranging impact of immune dysfunction on behavioral change in ASD patients. Detecting a convergent effect of immune function on neurodevelopment and behavioral symptoms in ASD should be the focus of future research.

### Strengths of the study:

The strength of this study is that the findings are based on an appropriate sample size which support the role of autoimmunity in the damage of neurons in CNS. This study also reported very important markers which play major role in autoimmunity process.

### Limitations of the study:

First, the search is limited to the findings associated only to Saudi autistic children reported by ARTC, KSU, Riyadh, KSA. Second, it is possible that we have omitted important studies by searching only English-language literature. This analysis was also limited by the heterogeneity among patient populations and outcomes of results. Therefore, the present review may not assess the findings appropriately; hence, further studies from other nations/ countries can be conducted to validate the results and clarify the role of autoimmunity in ASD.

## CONCLUSION

Autoimmunity plays a pathogenic role in CNS and etiologically important in ASD. Considerably greater values of autoantibodies are identified in ASD subjects as compared to healthy controls, however, the pathophysiological importance of these antibodies identified in ASD children is not well known. Overall, the results of autoimmunity in ASD families with excess of anti-brain antibodies suggest that in some patients, autoantibodies that target the CNS may be an aggravating or pathological factor in neuronal growth in autistic children. Large cohort studies with well-defined and specially pheno typed autistic groups and matched healthy controls are required to examine the role of autoantibodies in the pathology of subjects with ASD.

### Authors Contribution:

**DMH:** Data collection and manuscript writing and responsible and accountable for the accuracy or integrity of the work.

**LYA:** Review and final approval of manuscript.

**AMA:** Conceived, designed and editing of manuscript.

**NEE:** Data collection and editing.
